# Effects of Cationic Dendrimers and Their Complexes with microRNAs on Immunocompetent Cells

**DOI:** 10.3390/pharmaceutics15010148

**Published:** 2022-12-31

**Authors:** Nadezhda Knauer, Ekaterina Pashkina, Alina Aktanova, Olga Boeva, Valeria Arkhipova, Margarita Barkovskaya, Mariya Meschaninova, Andrii Karpus, Jean-Pierre Majoral, Vladimir Kozlov, Evgeny Apartsin

**Affiliations:** 1 Research Institute of Fundamental and Clinical Immunology, 630099 Novosibirsk, Russia; 2 Clinic for Neurosurgery, Medical Faculty and Heinrich-Heine University Medical Center Düsseldorf, 40225 Düsseldorf, Germany; 3 Institute of Chemical Biology and Fundamental Medicine SB RAS, 630090 Novosibirsk, Russia; 4 Laboratoire de Chimie de Coordination, CNRS, 205 Route de Narbonne, CEDEX 04, 31077 Toulouse, France

**Keywords:** dendrimers, microRNA, immunotherapy, nanomedicine, PBMCs, cytokines, surface markers

## Abstract

Short regulatory oligonucleotides are considered prospective tools for immunotherapy. However, they require an adequate carrier to deliver potential therapeutics into immune cells. Herein, we explore the potential of polycationic dendrimers as carriers for microRNAs in peripheral blood mononuclear cells of healthy donors. As an oligonucleotide cargo, we use a synthetic mimic and an inhibitor of miR-155, an important factor in the development and functioning of immunocompetent cells. Dendrimers bind microRNAs into low-cytotoxic polyelectrolyte complexes that are efficiently uptaken by immunocompetent cells. We have shown these complexes to affect the number of T-regulatory cells, CD14^+^ and CD19^+^ cell subpopulations in non-activated mononuclear cells. The treatment affected the expression of HLA-DR on T-cells and PD-1 expression on T- and B-lymphocytes. It also affected the production of IL-4 and IL-10, but not the perforin and granzyme B production. Our findings suggest the potential of dendrimer-mediated microRNA-155 treatment for immunotherapy, though the activity of microRNA-dendrimer constructions on distinct immune cell subsets can be further improved.

## 1. Introduction

Immunotherapy as a method of targeted modulation of the immune response became an important element of the therapeutic landscape nowadays [1,2]. However, modulating the activity of the immunocompetent cells, especially, cells which form the tumor microenvironment, could be another perspective way to work—it can allow not to catch every tumor cell escaping the immune response, but to make the process of antitumor response more effective [3].

Immune processes are mediated by many effectors and regulatory factors. This calls for multifunctional bioactive regulators to be used to treat immune cells. In particular, microRNAs (miRs) seem to be promising candidate entities. These small (ca. 18–25 nucleotides) non-coding RNAs are involved in many processes of cell life, being part of RNA-induced silencing complex (RISC), which suppresses mRNA translation [4,5,6]. Targeting more than one messenger RNA, a single miR may have various biological effects related to the modulation of genes’ expression [6,7]. Alternatively, desired effects can be reached by inhibiting existing cellular miRs using synthetic antagonist oligonucleotides [8,9,10]. Due to the pleiotropic effects, miR-based therapy can be a promising approach for immunotherapeutic protocols.

However, miR-based immunotherapy faces several limitations related to the low stability of oligonucleotides in biological media and their relatively low uptake by target cells. The former obstacle can be overcome by specific chemical modifications in oligonucleotides [8], whereas the latter frequently requires the use of carriers. In general, these carriers should be biocompatible and non-immunogenic (this feature can be optional, depending on the target); miR-loaded vehicles are expected to be efficiently uptaken by target cells releasing the miR cargo [3,11]. Modern techniques offer different types of vectors for miR delivery: liposomes, stable nucleic acid–lipid particles, “hard” nanoparticles, synthetic and natural polymers, etc. [8,9,11]. Among the abovementioned species, dendrimers—rationally designed synthetic polymers—are of particular interest.

Dendrimers are a family of highly symmetrical hyperbranched molecules of different chemical origins; they consist of a core and branches built of repeating units bearing branching points [12]. Optimizing the chemical structure and features of structural units, which is possible thanks to rich interior and surface chemistry, one can modulate the physicochemical properties of dendrimers as well as their biological behavior [13,14,15,16]. Moreover, their synthesis can be easily controlled and adding additional functional groups allows for modifying the properties of the molecule, which is important for potential commercial use [7,17]. These nanostructures are monodisperse and can be characterized as structures with precise molecular weight, which can be a great advantage for their commercial use [18].

Dendrimers containing main group elements in the core or as branching points have demonstrated excellent chemical stability and good biocompatibility acceptable for biomedical applications. Polycationic dendrimers of phosphorus-based or silicon-based architecture have been shown to serve as efficient transporters of therapeutic oligonucleotide constructions [12,19,20,21]. Their unique characteristics permit to achieve the transfection of highly complex biological objects, namely immune cells—a highly heterogenous population including cell types known to be poorly transfectable by commonly used agents.

Herein, we explore the effects of regulatory microRNAs on naïve peripheral blood mononuclear cells (PBMCs) of healthy donors upon delivery with polycationic phosphorus and carbosilane dendrimers. As a therapeutic microRNA, we have chosen miR-155, a well-described microRNA with immunostimulant properties, as well as its synthetical inhibitor [22,23], bound with dendrimers into polyelectrolyte complexes (dendriplexes).

## 2. Materials and Methods

### 2.1. Dendrimers

**Figure 1:**
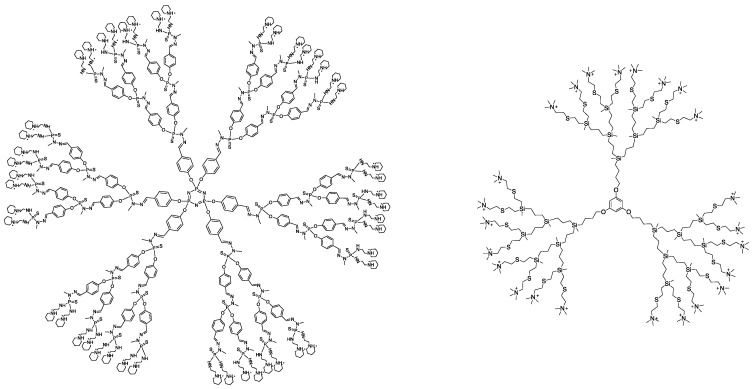
Molecular structures of the cationic phosphorus dendrimer AE2G3 (**left**) and carbosilane dendrimer BDEF33 (**right**) used in the study.

Polycationic phosphorus (AE2G3) and carbosilane (BDEF33) dendrimers were prepared and characterized according to the reported methods [12,24]. The molecular structures of the dendrimers are given in Figure 1.

Dendrimer AE2G3 was synthesized by functionalization of P(S)Cl_2_-terminated G3 precursor with aminoethyl piperidine in the presence of DIPEA as a base. The precursor, in turn, was obtained based on hexachlorocyclotriphosphazene by iterative reactions with hydroxybenzaldehyde and dichlorothiophosphomethylhydrazine.

Dendrimer BDEF33 was obtained from the vinyl-terminated precursor dendrimer by thiol-ene reaction with dimethylaminoethylthiol followed by quaternization with methyl iodide. The precursor dendrimer was obtained by grafting of vinyl-terminated G3 dendrons onto phloroglucinol core. G3 dendrons were synthesized based on bromobutyl-methyldichlorosilane by iterative Grignard and hydrosilylation reactions.

It is worth noting that using two orthogonal reactions for constructing dendrimer scaffold it is possible to obtain monodisperse macromolecular products instead of polydisperse hyperbranched polymers.

### 2.2. microRNAs

The synthesis of microRNA miR-155: 5′-r(UUAAUGCUAAUCGUGAUAGGGGUU) and its antagonist amiR-155: 5′-m(ACCCCUAUCACGAUUAGCAUUAA) (r—ribonucleotides; m—2′-O-methylribonucleotides) was carried out on an automatic ASM-800 DNA/RNA synthesizer (Biosset, Novosibirsk, Russia) in the 0.4 µmol scale with the use of 2′-O-TBDMS-protected RNA phosphoramidites (5-(ethylthio)-1H-tetrazole as an activator, coupling time was 5 min) and the automated procedures optimized for the synthesizer [25].

### 2.3. Therapeutic Formulations

Dendriplexes were formed by combining oligonucleotides and dendrimers AE2G3 or BDEF33 (see concentrations in Table 1) in the RNase free 1× PBS buffer (10 mM phosphate buffer, pH 7.4, 137 mM NaCl, 2.7 mM KCl) with the following incubation for 10 min at 25 °C.

Characteristics of dendriplexes were obtained using Zetasizer Nano ZS particle analyzer (Malvern Instruments, Manchester, UK), equipped with NBS. The measurements were made at 25 °C.

### 2.4. Blood Samples

The samples of venous blood from healthy donors (descriptions for every group are given Supplementary) were used in the study. Informed written consent was obtained from all donors. The study design as well as the present manuscript were approved by the Institutional Local Ethical Committee of the Research Institute of Fundamental and Clinical Immunology (protocol No. 140, 23 September 2022).

### 2.5. Preparation of the Peripheral Blood Mononuclear Cells (PBMCs)

Peripheral blood mononuclear cells (PBMCs) were extracted from the heparinized blood (10 mL) on the ficoll-urografin gradient (1077 mg/mL) according to the standard procedures [26]. Cells were cultivated in the RPMI-1640 cell medium (PanEco, Moscow, Russia) with 10% fetal calf serum (Thermo Fisher Scientific, Waltham, MA, USA) with the addition of molecules under study.

### 2.6. Internalization of miR-Containing Complexes

Cells (*n* = 6, see Supplementary) were cultivated in 12-wells flat-bottom plates (100,000 cells in 500 µL total volume) in presence of BDEF33 or AE2G3 complexes with amiR-155-FAM (100 nM in RNA equivalent according to the charge ratio), non-treated cells were used as a control (NTC). Cells were incubated in standard conditions (humidified 37 °C, 5% CO_2_) for 4 h then collected, and washed twice by cold PBS (Thermo Fisher Scientific, Waltham, MA, USA) containing 10% FBS (Thermo Fisher Scientific, Waltham, MA, USA) and 2 mM EDTA (ethylenediaminetetraacetic acid, Sigma-Aldrich, St. Louis, MO, USA). To remove molecules being stuck on the surface, treatment by acidic glycine buffer (50 mM, pH 3.0) was performed after the first washing. Cells were fixed by 4% PFA solution (paraformaldehyde, Sigma-Aldrich) and analyzed. Flow cytometry analysis was performed on FACS Canto II (BD, Piscataway, NJ, USA) machine by using FACS Diva (BD, Piscataway, NJ, USA) software.

### 2.7. Fluorescence Microscopy

Internalization of fluorescently labeled dendriplexes was investigated by fluorescence microscopy. Cells were incubated with BDEF33 or AE2G3 complexes with or without amiR-155-FAM and alone as stated above. Then, cells were washed twice with 2 mM EDTA solution in PBS and fixed with a mixture of ethanol:glacial acetic acid (3:1, *v*/*v*). Cells were resuspended thoroughly to avoid clumps after fixation step and placed on slides. 

Then, the slides were analyzed using phase-contrast microscopy for cell cytoplasm and nuclei visualization in transmitted light. After that, slides were mounted with Pro Long Gold antifade containing DAPI (Invitrogen MP, Waltham, MA, USA) to prevent dye photo-bleaching and identify cell nuclei further.

Phase-contrast, as well as fluorescent microscopy, was performed with the Axioscope 40 fluorescence microscope (Zeiss, Germany) equipped with a high-pressure mercury lamp HBO 50W, with Zeiss interference filter sets (Set No. 49 for DAPI) and CCD-chamber AxioCam 503 mono (at 1936 × 1460 px resolution and 14-bit capacity). DAPI-stained nuclei images and FAM signals were captured separately with the software package ZEN-2012 (Zeiss, Germany) on the magnitude X1000 (Figure S1). Exposure time was adjusted automatically. 

### 2.8. WST-Assay

For evaluation of cell viability, we performed a WST assay; the number of donors in every group varied from 5 to 8 (see Supplementary), and all the samples were processed in 2 technical replicates. PBMCs were seeded at 96-well flat-bottomed culture plates (100,000 cells in 100 µL total volume), treated by free dendrimers (BDEF33 or AE2G3; 0 µM; 0.3 µM; 1 µM; 3 µM; 10 µM; 30 µM) or dendriplexes, containing miR-155 or its synthetic antagonist amiR-155 (25 nM, 50 nM, 100 nM, 150 nM in RNA equivalent according to the charge ratio; or individual components in appropriate concentrations), then incubated for 72 h in standard conditions (humidified 37 °C, 5% CO_2_). Non-treated cells were used as a control (non-treated control, NTC).

For performing WST assay 10 µL of WST-1 reagent (Premix WST-1 Cell Proliferation Assay System, Takara Bio, Kusatsu-shi, Japan) were added to every well and mixed thoroughly, plates were incubated for 4 h in the CO_2_-incubator. Then, absorbances at 450 nm and 620 nm were read on a microplate reader (Infinite F50, Tecan, Grödig, Austria).

### 2.9. LDH Activity Assay

We evaluated LDH activity in accordance with the recommendations of the developer (LDH Assay Kit Colorimetric, Abcam, Boston, MA, USA). Cells (10^6^/mL) were cultured with samples at the same concentrations and conditions as the Apoptosis assay (*n* = 5, see Supplementary). After cultivation, cells were washed with cold PBS, homogenized on ice in 2–4 volumes of cold Assay Buffer and centrifuged at 4 °C at 10,000× *g* for 15 min in a cold microcentrifuge to remove any insoluble material from samples. We choose 10 min and 20 min as time-points to evaluate the enzyme activity after the addition of the LDH reaction mix. The optical density was read with Infinite F50 (Tecan, Grödig, Austria, Gmbh) microplate reader set at 450 nm with a 620 nm reference wavelength.

### 2.10. Apoptosis Assay

A group of donors (*n* = 6, see Supplementary) were used to evaluate apoptosis in freshly isolated PBMCs. The determination of apoptosis was performed in accordance with the instructions for the FITC Annexin V Apoptosis Detection Kit with PI (BioLegend, San Diego, CA, USA). The cells at a concentration of 5 × 10^5^ cells/mL were incubated in 12-well plates with free dendrimers (BDEF33 or AE2G3; 10 µM) or dendriplexes, containing miR-155, its synthetic antagonist amiR-155 (150 nM in RNA equivalent according to the charge ratio; or individual components in appropriate concentrations), then incubated for 72 h in standard conditions (humidified 37 °C, 5% CO_2_). After cultivation, the cells were collected and labeled with surface markers. Then, PBMCs were washed with Annexin binding buffer and resuspended in 100 μL of Annexin-V (AnV) and PI dual-stain solution (0.1 μg of Annexin-V FITC and 1 μg of PI) for 15 min in the dark. After adding Annexin binding buffer, cells were analyzed by a FACSCanto II flow cytometer (Becton Dickenson, Franklin Lakes, NJ, USA) using FACSDiva 6.1 software APC (Becton Dickenson, Franklin Lakes, NJ, USA). The toxic effect of samples on the population of T-lymphocytes (CD3^+^/CD4^+^; CD3^+^/CD4^−^) of donors was established according to the level of AnV^+^/PI^−^ early apoptotic cells and AnV^+^/PI^+^ late apoptotic/necrotic cells.

### 2.11. Expression of Surface Molecules

For performing flow cytometry, we used fluorescent-labeled monoclonal antibodies to particular surface molecules: CD45-APC/Cy7, CD3-PerCP, CD3-PE/Cy7, CD4-APC, CD4-APC/Cy7, CD8-PE/Cy7, CD25-APC, CD127-PerCP/Cy 5.5, CD14-PE/Cy7, CD19-APC, CD16-PerCP, CD56-PE/Cy7 (all Biolegend, San Diego, CA, USA). CD25-PE and HLA-DR-APC/Cy7 antibodies (Biolegend, San Diego, CA, USA) were used for evaluating of the expression of activation markers. Flow cytometry analysis was performed on FACS Canto II (BD, Piscataway, NJ, USA) machine by using FACS Diva (BD, Piscataway, NJ, USA) software. We used non-stained controls, FMO controls, and isotypic controls.

### 2.12. Cell Activation and Proliferation Assay

The PBMCs from 6 donors were used in this assay. Cells were treated with dendrimers and dendriplexes were studied for 72 h, non-treated cells were used as a control. The proliferation of PBMCs was assessed by flow cytometry of carboxyfluorescein succinimidyl ester (CFSE) stained cells. Cells were labeled before culturing with 5,6-carboxyfluorescein diacetate succinimidyl ester (CFSE) (4 μM) (Invitrogen, Eugene, OR, USA), mixed well and incubated for 15 min in darkness, stirring occasionally. Then, the reaction was stopped by adding 2 mL of PBS with 5% FCS, followed by centrifugation. To evaluate the proliferation of lymphocyte subsets, PBMCs after cultivation were stained with monoclonal anti-human antibodies (CD45-APC/Cy7, CD3-PE/Cy7, CD4-APC, CD25-PE, HLA-DR-APC/Cy7, CD127-PerCP/Cy5.5, or CD45-APC/Cy7, CD14-PE, CD19-APC, CD16-PerCP, CD56-PE/Cy7). Analyses were performed using FACSCanto II (Becton Dickinson, Franklin Lakes, NJ, USA) and FACSDiva software (Becton Dickinson, Franklin Lakes, NJ, USA).

### 2.13. Evaluation of Perforin and Granzyme B Production

Cells (*n* = 6, see Supplementary) were cultivated for 72 h in the presence or absence of dendrimers and dendriplexes. After cultivation, cells were surface stained with CD3-APC, CD8-PE/Cy7, CD16-PerCP, and CD56-APC/Cy7 antibodies. Cells were then fixed (Cytofix; BD Biosciences), followed by intracellular staining (granzyme B-PE, perforin-FITC) as indicated in permeabilization solution (Cytoperm; BD Biosciences). A set of isotype control antibodies were used as a control for the intracellular staining.

### 2.14. Cytokine Secretion Analysis

We used ELISA to evaluate the secretion of IL-10, IL-4, TNF alpha and IFN gamma according to the manufacturer`s protocols (all kits were from Vector Best, Novosibirsk, Russia) (*n* = 5, see Supplementary). The values measured were expressed as a percentage of the value of NTC for every donor to eliminate interpersonal deviations.

### 2.15. Statistical Analysis

We used STATISTICA for Windows 10 (StatSoft) and GraphPad Prism 7 (GraphPad Software) software for statistical analysis and data visualization. Mann-Whitney test was used, *p*-value < 0.05 is considered statistically significant.

## 3. Results

### 3.1. BDEF33 and AE2G3 Dendrimers Efficiently Transport microRNA into the Cells

Polycationic dendrimers efficiently bind both oligonucleotides into nanoscale polyelectrolyte complexes (dendriplexes). The complexation protocols were optimized in such a way as to fully bind RNA in samples and saturate it with cationic carriers [19]. The dendriplexes formed usually have a spherical shape and compact morphology [20,27]. The size of dendriplexes (30–50 nm) as well as their charge (cationic for AE2G3-containing ones and slightly cationic for BDEF33-containing ones) (Table 2). These parameters are considered optimal for efficient cell uptake. Importantly, we have recently shown that AE2G3 and BDEF33 efficiently protect RNA from nuclease digestion [21].

**Figure 2:**
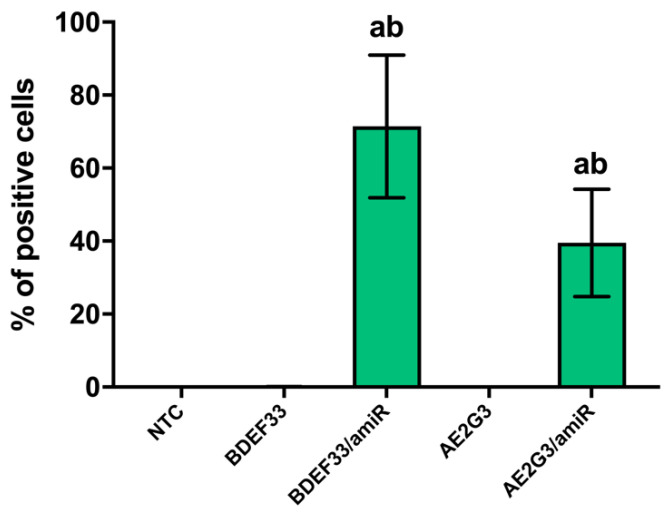
Evaluation of internalization of dendriplexes containing fluorescent-labeled RNA (amiR-155-FAM) into PBMCs (*n* = 3) after 4 h of treatment. Mann–Whitney test was used. Letter “a” marks significant differences (*p* < 0.05) in comparison with NTC, “b” marks significant differences in comparison with a free dendrimer.

We found that microRNA-dendrimer complexes can be efficiently internalized into PBMCs (see Figure 2), either BDEF33-based complexes (*p* = 0.00313 in comparison with NTC) or AE2G3-based complexes (*p* = 0.0078 in comparison with NTC). A slight difference between the two types of dendriplexes was observed (*p* = 0.06). Fluorescent microscopy images confirmed these results (see Figure S1).

### 3.2. BDEF33- and AE2G3-Based Dendriplexes Did Not Demonstrate Significant Toxicity against PBMCs in General and against T-Cell Subsets Particularly

We have formed dendriplexes using dendrimers in concentrations (see Table 1) that are lower than the IC50 of both molecules (see Figure S2). We did not observe any significant toxic effects neither for BDEF33-based complexes, nor for AE2G3-based complexes on PBMCs analyzing WST-1 data (see Figure S3), results of LDH activity assay (see Figure S4), nor parameters of apoptosis induction for CD4^+^ and CD8^+^ lymphocytes (see Figure S5). That allows us to conclude that dendriplexes under study have high biocompatibility characteristics and low toxic effects in this cell model.

### 3.3. Treatment by Dendriplexes Did Not Change Neither T-Cells Subsets Ratio Nor Their Proliferative Activity, but Changes in the Number of T-Regulatory Cells, CD14^+^ and CD19^+^ Cells Were Found

**Figure 3:**
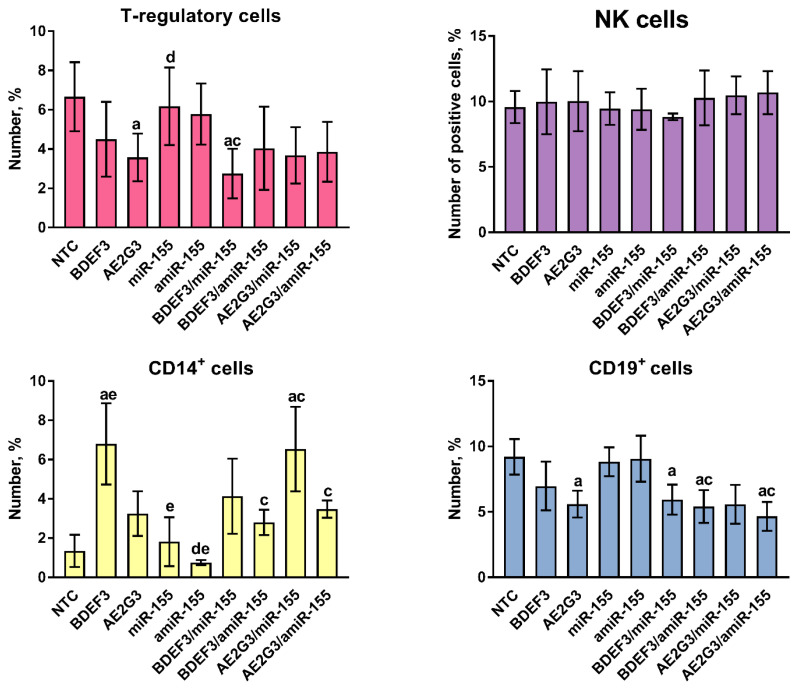
Evaluation of subsets percentage after the treatment by dendriplexes or their components (72 h): T-regulatory cells (CD3^+^CD4^+^CD25^+^CD127^low^), NK-cells (CD16^+^CD56^+^), B-lymphocytes (CD45^+^CD19^+^). Mann–Whitney test was used. Letters mark significant differences (*p* < 0.05): “a”—in comparison with NTC, “c”—in comparison with treatment by free miR, “d”—in comparison with treatment by BDEF33-based complex, “e”—in comparison with by AE2G3-based complex.

In the first step, we evaluated the number of several subsets of immunocompetent cells: CD3^+^, CD4^+^, CD8^+^, T-regulatory cells (CD3^+^CD4^+^CD25^+^CD127^low^), NK-cells (CD16^+^CD56^+^) (see Figure 3). The amount of CD3^+^ cells did not change significantly. We found just a slight increase in CD3^+^ cells after treatment by AE2G3, AE2G3/miR-155, AE2G3/amiR-155 (*p* = 0.068 for all cases) in comparison with NTC (see Figure S6). At the same time, no significant difference in T-cells subsets proliferation was observed (see Figure S7).

BDEF33 treatment decreased the number of T-regulatory cells in comparison with NTC (*p* = 0.043), the same for AE2G3 (*p* = 0.059). Treatment by BDEF33/miR-155 dendriplex also decreased the number of T-regulatory cells in comparison with NTC (*p* = 0.028), similar for BDEF33/amiR-155 (*p* = 0.063), BDEF33/NC (*p* = 0.067). AE2G3/amiR-155 treatment slightly decreased Treg numbers (*p* = 0.063).

Free miR-155 demonstrated lower suppressive effect on Treg number than BDEF33/miR-155 (*p* = 0.016) and AE2G3/miR-155 (*p* = 0.063).

Treatment by BDEF33 and AE2G3 led to increasing of CD14^+^ cells number (*p* = 0.062 and 0.031, respectively). Same effect was observed after treatment by AE2G3/miR-155 (*p* = 0.03) and AE2G3/NC (*p* = 0.031) complexes. Free miRs had lower activity than dendriplexes: miR-155 in comparison with AE2G3/miR-155 (*p* = 0.014); amiR-155 in comparison with BDEF33/amiR-155 (*p* = 0.0079) and AE2G3/amiR-155 (*p* = 0.014).

A slight increase in NK cell number was found after treatment by AE2G3/miR-155 (*p* = 0.063) and AE2G3/amiR-155 (*p* = 0.063) compared to NTC.

A number of CD45^+^CD19^+^ B-lymphocytes were observed to be lower in comparison with NTC after treatment by free AE2G3 (0.06) and BDEF33 (0.031). Similar effects were found for dendriplexes (*p* = 0.031 for AE2G3/miR-155, AE2G3/amiR-155, BDEF33/amiR-155 and *p* = 0.063 for BDEF33/miR-155).

### 3.4. Expression of CD25+ on T-Cells Was Intact after Treatment but Changes in HLA-DR Expression Were Observed

The expression on CD25 did not change significantly, nor on CD4^+^ or on CD8^+^ T-cells (see Figure S8). At the same time, we found that treatment by AE2G3/miR-155 complexes increased HLA-DR expression on CD4^+^ cells compared to NTC (*p* = 0.0313) and similar complex BDEF33/miR-155 (*p* = 0.03).

**Figure 4:**
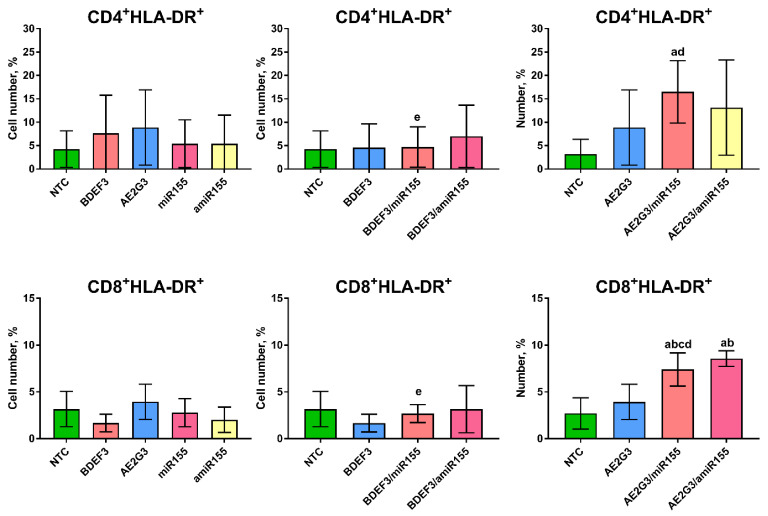
Evaluation of expression of HLA-DR activation marker on CD4+ and CD8+ T-lymphocytes after 72 h of treatment by dendriplexes or their complexes. Mann–Whitney test was used. Letters mark significant differences (*p* < 0.05): “a”—in comparison with NTC, “b”—in comparison with treatment by free dendrimer, “c”—in comparison with treatment by free miR, “d”—in comparison with treatment by BDEF33-based complex, “e”—in comparison with by AE2G3-based complex.

HLA-DR expression on CD8+ cells (See Figure 4) was observed to be increased after treatment by AE2G3/miR-155 in comparison with NTC (*p* = 0.021), with free AE2G3 (*p* = 0.06), free miR-155 (*p* = 0.0087) and BDEF33/miR-155 complex (*p* = 0.0313). Similar effect was found for AE2G3/amiR-155 complex comparing to NTC (*p* = 0.03), to AE2G3 (*p* = 0.002).

### 3.5. Dendriplexes and Their Components Can Change PD-1 Expression on T- and B-Lymphocytes

**Figure 5:**
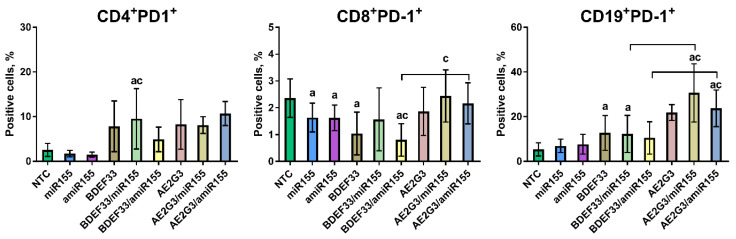
Evaluation of expression of PD-1 on CD4^+^ and CD8^+^ T-lymphocytes, CD19^+^ B-lymphocytes after 72 h of treatment by dendriplexes or their complexes. Mann–Whitney test was used. Letters mark significant differences (*p* < 0.05): “a”—in comparison with NTC, “c”—in comparison with treatment by free miR, bars mark the difference between similar groups.

Investigating the PD-1 expression on effector cells after 72 h of treatment, we observed different patterns (see Figure 5).

For CD4^+^ T-lymphocytes this parameter did not change significantly after treatment by free miRs or by free dendrimers (mock controls). At the same time using of dendriplexes slightly increased PD-1 expression in comparison with NTC (*p* = 0.046 for BDEF33/miR-155 and *p* = 0.06 for both AE2G3 complexes). Moreover, complexes increased PD-1 expression in comparison with free miRs (*p* = 0.016 for the BDEF33/miR-155 complex and *p* = 0.06 for both AE2G3 complexes).

On CD8^+^ T-lymphocytes PD-1 expression was found to be decreased after treatment by free miRs (*p* = 0.016 for miR-155, *p* = 0.031 for amiR-155), free BDEF33 (*p* = 0.016) and BDEF33/amiR-155 complex (*p* = 0.016) in comparison with NTC. miR-155 demonstrated higher effect in comparison with AE2G3/miR-155 complex (*p* = 0.047), and amiR-155—comparing to BDFE33/amiR-155 complex (*p* = 0.031). BDEF33/amiR-155 complex decreased PD-1 expression more actively than AE2G3/amiR-155 complex (*p* = 0.03).

In terms of CD19^+^ B-lymphocytes, both types of miRs did not change PD-1 expression significantly. AE2G3 and its complexes increased this parameter (*p* = 0.06 for free AE2G3 and 0.03 for both complexes), and the same effect was observed for the BDEF33 group (*p* = 0.31 for free BDEF33 and BDEF33/miR-155 complex and 0.06 for BDEF33/amiR-155). Both AE2G3/miR complexes had significantly higher activity compared to free miRs (*p* = 0.031). BDEF33 complexes had a lower effect than AE2G3-based dendriplexes (*p* = 0.03 for both).

### 3.6. Dendriplexes Changed Production of IL-4 and IL-10, but Not the Perforin and Granzyme B Production

In the process of evaluation of the functional activity of immunocompetent cells, we did not find any significant effects of free dendrimers, free miRs or dendriplexes on granzyme B and perforin production by CD8+ T-lymphocytes or by NK-cells (see Figure S9).

**Figure 6:**
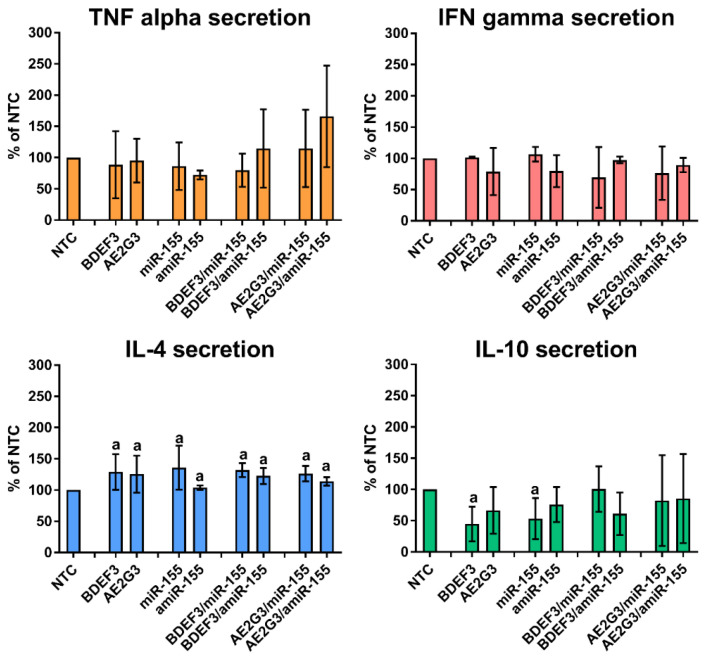
Evaluation of secretion of cytokines after 72 h of treatment by dendriplexes and their free complexes, being expressed as percentage of values measured for non-treated control (NTC). Mann–Whitney test was used. Letter “a” marks significant differences (*p* < 0.05) in comparison with NTC.

We also did not observe any significant changes in the secretion of TNF alpha and IFN gamma (see Figure 6).

Secretion of IL-4 increased after treatment by free dendrimers (*p* = 0.007 for both BDEF33 and AE2G3), and free miRs (*p* = 0.03). Adding of dendriplexes also led to higher IL-4 expression, either for BDEF33 complexes (BDEF33/miR-155, *p* = 0.027; BDEF33/amiR-155, *p* = 0.0047; BDEF33/NC, *p* = 0.0027) or for AE2G3 (AE2G3/miR-155, *p* = 0.008; AE2G3/amiR-155, *p* = 0.005; AE2G3/NC, *p* = 0.005).

Regarding IL-10 secretion, only a slight decrease was found after treatment by BDEF33 (*p* = 0.0313), and miR-155 (*p* = 0.0313).

## 4. Discussion

Nowadays, microRNAs are supposed to be one of the most promising instruments for immunomodulatory and antitumor therapy. Their function as regulators of posttranslational gene regulation and pleiotropic activities allows the modulation of gene expression and manages the processes in the cells [9]. Since miRs are involved in different pathways of development of numerous pathological conditions, the use of their mimics or antagonists can help in treating diseases [7,22].

Previous studies mostly focused on the use of therapeutic oligonucleotides to deliver into the tumor cells but the targeting of these substances to immunocompetent cells can be another interesting approach. We choose microRNA-155—one of the most well-known immunomodulatory miR—and its synthetic inhibitor for the study, as agents whose delivery is of great scientific interest [23,28].

miR-155 plays an important role in different steps of developing and functioning of immunocompetent cells. This RNA, the product of a BIC gene transcript, belongs to the miRs, which expression is highly specific for hematopoietic cells (in murine models) [23]. Expression of miR-155 is increasing during activation of immunocompetent cells—T and B lymphocytes, macrophages and dendritic cells; it is involved in the process of IFNγ production by NK-cells after their activation [29,30]. Acetylation of the H3 histone of the miR-155 gene leads to enhancing its transcription and silencing of B-cells genes, involved in DNA recombination during class switching, and the process of somatic hypermutation [22,30].

The use of nucleic acids as therapeutic instruments meets several difficulties to be solved: risk of enzymatic degradation, rapid clearance and low cell membrane permeability [7,9,19]. To overcome these obstacles, nanosized carriers can be used. In particular, soft nanoparticles generally have low immunogenicity and high capacity, they are easy-to-made materials, but at the same time, their use can have some disadvantages related to cytotoxic effects or low transfection efficiency [8,9,31].

Another problem that appears in this process is that parameters of cell permeability for the nanoparticles were mostly studied on tumor models or models related to inflammatory processes [9,32]. These conditions have a positive effect on particles uptake (for example, because of the so-called Enhanced Permeability and Retention effect) with some limitations [2,31,33], however, if we switch to immunocompetent cells, and T-cells especially, this task becomes more complicated—parameters of uptake strongly depend on the subset and its functional activity [34,35,36]. At the same time, targeting cells providing adaptive and/or innate immunity (in contrast to avoiding interactions between nanoparticles and immunocompetent cells) can be a really promising approach for the needs of modern immunotherapy [3,37].

Thus, we need a vehicle with a high loading capacity, great protective properties, and low toxicity. Dendrimers have already shown their efficacy in different models, their effects on immunocompetent cells were related mostly to the anti-inflammatory shift: suppressing expressions of genes, which are involved in the inflammatory response, decreasing IL-12, TNFα, IL-6 secretion and higher IL-10 secretion, induction of dendritic cells with the tolerogenic phenotype (it should be noted that concentrations of dendrimers in these experiments were higher than in current study) [38,39,40,41,42,43,44,45] Additionally, their efficacy as carriers of therapeutic nucleic acids was proven in numerous previous studies on different cell models [18,19,27,46,47,48]. On the other side, it should be noted that the dendrimer molecules studied have their own toxic effect even in low and medium concentrations [18]. It is supposed to depend not only on the chemical origin of the molecule but also on its generation and molecular weight, which is typical for all similar polymers [7]. On contrary, dendrimers of higher generations usually have better penetrative activity [19], so the balance between biocompatibility and transport efficacy has to be found.

In our study, we also observed this cytotoxic effect of AE2G3 and BDEF33 per se, and the IC50 of the carbosilane dendrimer BDEF33 was much higher than for phosphorus dendrimer AE2G3. This activity is more “preferable” in studies of anti-tumor therapeutics (which are supposed to be cytotoxic), but we should note that in our previous works, tumor cells (models of leukemia) were observed to be more sensitive to dendrimers than PBMCs (publication in preparation). It can be related to the different activity of intracellular uptake of tumor and non-tumor cells.

The concentrations of dendrimers used for the preparation of complexes with oligonucleotides (dendriplexes) calculated according to the optimal charge ratio are significantly lower than IC50, so they should have lower cytotoxicity. Additionally, it was proven by the fact that neither AE2G3-, nor BDEF33-based complexes with miR-155 or its antagonist did not demonstrate significant toxic effects on WST tests or after evaluation of LDH activity regarding PBMCs culture; they did not induce apoptosis in CD4^+^ and CD8^+^ T-cells. Therefore, we can take up the position that dendriplexes are substances with high biocompatibility values in this model.

Another parameter that is critically important for the molecular vehicle is its penetrating activity allowing to transport the of therapeutic substances into the cells. We demonstrated that dendrimers could be efficient carriers of microRNAs (see Section 3.1) Similar results were also observed earlier for siRNAs being delivered into glioblastoma stem-like cells, HeLa cells and induced pluripotent stem cells, moreover, dendrimer transport activity was higher than for Lipofectamine 2000 and Lipofectamine 3000 [18,21].

Regarding parameters of phenotypical characteristics of cells treated by dendriplexes or their complexes and their impact on proliferative activity, the effect observed was rather slight. Neither CD4^+^ nor CD8^+^ cells, as well as NK cells, did not change their number or proliferative activity (see Section 3.3). Free miR-155 did not change the amount of T-regulatory cells significantly, but at the same time, free BDEF33 and its complex BDEF33/miR-155 demonstrated a slight suppressive effect on T-regulatory cell number. In the case of CD14^+^ cells, AE2G3 and its complexes showed a positive effect on the cell number and the CD19^+^ cell treatment by both dendrimers and their complexes had a slightly negative effect. This decrease in the number of specific subsets of immunocompetent cells is rather controversial in comparison with the data about the immunostimulatory effects of miR-155, but probably it can be explained by the selective sensitivity of different cell types to dendrimers, for example, due to different permeability of cell membrane. Intriguingly, choosing the microRNA (miR-155 or its inhibitor) did not change the effect significantly.

On the next step, the expression of activation markers on T-cells subsets was evaluated. We hypothesized that using microRNA with activating properties or its complexes can lead to higher expression of the markers, but surprisingly CD4^+^ cells did not respond, only the increase in HLA-DR expression on CD8^+^ cells was shown (see Section 3.4). This effect was related to AE2G3 complexes with miR-155 and its inhibitor.

As we noticed earlier neither the amount nor proliferation of CD4^+^ and CD8^+^ cells did not change after treatment. However, such parameter as the expression of PD-1 was modulated—CD4^+^ cells were almost intact, at the same time the CD8^+^ cells responded: using both types of RNAs, BDEF and BDEF-based dendriplex led to decreasing of PD-L1 expression (see Section 3.5). In contrast, PD-1 expression increased in CD19^+^ cells, which also contradicts the fact of a lower number of CD19^+^ subset after treatment. We can probably hypothesize that CD19^+^ can internalize particles more actively, which provides a higher toxic effect on them (less cell amount); changing of PD-1 expression can be associated with involving of PD-1-PD-L1 axis into the metabolic regulation and oxidative cell stress [49,50].

Regarding functional activity, changes in perforin /granzyme B expression and cytokine-related response were supposed to be observed, according to the data describing the effects of miR-155 on these parameters [30,51]. At the same time, the changes, been found, were slight or even absent. Even CD8^+^ T-cells, which demonstrated modulation of HLA-DR and PD-1 expression, stayed intact.

Regarding the overall effects of microRNA-dendrimer complexes on immunocompetent cells, we constate that these effects were found to be unexpectedly low, especially, in T-cells. The most probable explanation is that we used non-activated cells, trying to describe the “pure” effect of nanoformulations. At the same time, it was shown that activated lymphocytes can incorporate nanoparticles more actively; interestingly, this parameter probably depends on the phase of the cell cycle [35,52,53]. Decorating dendrimers with functional groups targeting nanoformulations into immune cell subpopulations of interest [34] can probably help to overcome this obstacle.

## 5. Conclusions

Summarizing the results, we can presume that even though dendrimers can form complexes with therapeutic miRs and efficiently internalize them into the cells, the overall immunomodulatory effects of dendriplexes were lower than expected. This can be explained by the fact that we used non-activated immune cells to describe the “pure” effect of nanoformulations on PBMCs in blood. We have also found that dendritic molecules under study have their own activity even in low and medium concentrations. These effects were already observed in different previous studies which used cancer and non-cancer cells as models [18,19]. Thus, dendrimers could be a perspective platform for RNA delivery, at the same time, choosing of transporting molecules and the necessity of chemical modifications to improve transport have to be discussed.

## Figures and Tables

**Table 1:** Concentrations of complex components being used in the study.

Concentration of RNA, nM	Cation Excess	Concentration of BDEF33, μM	Concentration of AE2G3, μM
25	10	0.22	0.11
50	10	0.44	0.22
100	10	0.88	0.44
150	10	1.31	0.66

**Table 2:** Characteristics of dendriplexes obtained.

Dendriplex	Hydrodynamic Diameter (nm)	Zeta Potential (mV)	PDI
AE2G3	7.4 ± 0.9	+19.5 ± 1.1	0.18
AE2G3/miR-155	48.6 ± 1.6	+13.8 ± 0.3	0.22
AE2G3/anti-miR-155	45.2 ± 2.6	+14.0 ± 0.5	0.21
BDEF33	3.5 ± 0.5	+7.2 ± 1.4	0.19
BDEF33/miR-155	36.7 ± 5.2	+1.2 ± 0.2	0.25
BDEF33/anti-miR-155	40.4 ± 5.6	+1.5 ± 0.4	0.23

## Data Availability

Not applicable.

## Supplementary Materials

The supporting information can be downloaded at: https://www.mdpi.com/article/10.3390/pharmaceutics15010148/s1, Characteristics of donor groups; MTT and LDH assays results; FACS results. Figure S1: Microscopy images of cells treated with dendrimers, microRNA and dendriplexes. Magnification 400× Figure S2: Evaluation of proper toxic effects of cationic carbosilane (BDEF33) and phosphorus (AE2G3) dendrimers of the third generation on PBMCs viability in comparison with non-treated control cells (NTC). Mann-Whitney test was used. Letters mark significant differences (*p* < 0.05): “a”—in comparison with NTC, “b”—in comparison with treatment in concentration 0.3 µM, “c”—in comparison with treatment in concentration 1 µM, “d”—in comparison with treatment in concentration 3 µM, “e”—in comparison with treatment in concentration 10 µM, “f”—in comparison with treatment in concentration 30 µM. Bar show the significant difference between two variants of complexes; Figure S3: Evaluation of dendriplexes toxicity in comparison with non-treated control (NTC) and mock control (free dendrimer in the highest concentration being used—1.31 µM for BDEF33 and 0.66 µM for AE2G3). Mann-Whitney test was used; Figure S4: Evaluation of LDH activity after dendriplexes treatment (10 min, 20 min) in comparison with non-treated control (NTC), free miRs and free dendrimers. Mann-Whitney test was used. Letters mark significant differences (*p* < 0.05) between groups: “a”—in comparison with NTC; “b”—in comparison with a free dendrimer; “c”—in comparison with a free miR; “d”—in comparison with similar BDEF33-based complex; “e”—in comparison with similar AE2G3-based complex. Bars show the significant differences between columns in the subgroups; Figure S5: Evaluation of apoptosis parameters in CD4+ and CD8+ subsets after treatment by free dendrimers, free miRs or dendriplexes in comparison with non-treated control (NTC). Mann-Whitney test was used; Figure S6: Evaluation of subsets percentage after the treatment by dendriplexes or their components (72 h): CD3^+^, CD4^+^, CD8^+^. Mann-Whitney test was used; Figure S7: Evaluation of T-cells subsets proliferation after the treatment by dendriplexes or their components (72 h). Mann-Whitney test was used; Figure S8: Evaluation of expression of CD25 activation marker on CD4^+^ and CD8^+^ T-lymphocytes after 72 h of treatment by dendriplexes or their complexes. Mann-Whitney test was used. Letters mark significant differences (*p* < 0.05): “a”—in comparison with NTC, “b”—in comparison with treatment by free dendrimer, “c”—in comparison with treatment by free miR, “d”—in comparison with treatment by BDEF33-based complex, “e”—in comparison with by AE2G3-based complex; Figure S9: Evaluation of expression of granzyme B (GrzB) and perforin (Perf) by cytotoxic CD8^+^ T-lymphocytes and NK-cells (CD16^+^CD56^+^) after treatment by dendriplexes or their components. Mann-Whitney test was used; Table S1: Characteristics of donor groups under study.
