# Supplementary material for: Effects of Cationic Dendrimers and Their Complexes with microRNAs on Immunocompetent Cells
Source: Pharmaceutics. 2022 Dec 31;15(1):148. doi: 10.3390/pharmaceutics15010148 (PMC9862986; doi:10.3390/pharmaceutics15010148)

Supplementary Materials: Effects of Cationic Dendrimers and Their Complexes with microRNAs on Immunocompetent Cells

Nadezhda Knauer, Ekaterina Pashkina, Alina Aktanova, Olga Boeva, Valeria Arkhipova, Margarita Barkovskaya, Mariya Meschaninova, Andrii Karpus, Jean-Pierre Majoral, Vladimir Kozlov and Evgeny Apartsin

Table S1. Characteristics of donor groups under study.

Experiment:	N	M:F	Age (Mean ± SD)
WST assay:			
AE2G3	6	3:3	25.7 ± 1.6
AE2G3/miR	5	3:2	25.0 ± 0.0
BDEF33	5	1:4	37.2 ± 13.0
BDEF33/miR	8	3:5	25.3 ± 1.7
Apoptosis assay	6	2:4	29.0 ± 7.5
LDH assay	5	2:3	41.6 ± 21.5
Internalization	6	3:3	31.7 ± 9.9
Cells proliferation (CFSE-assay) and activation status	6	2:4	38.5 ± 20.9
Perforin and granzyme B production	6	2:4	27.5 ± 7.0
Cytokine secretion	5	1:4	41.2 ± 21.9

Figure S1. Microscopy images of cells treated with dendrimers, microRNA and dendriplexes. Magnification **400x.**

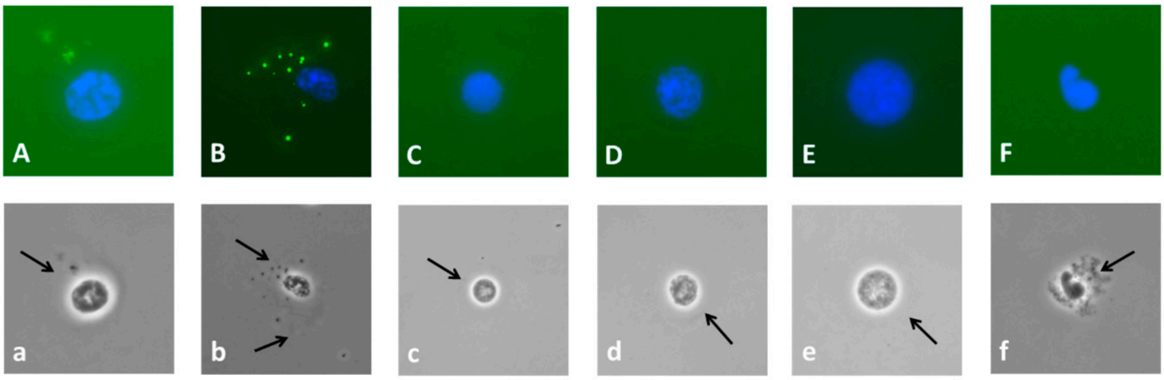

Note: Upper cases **(A-F)** represent merged fluorescent images on the magnitude X1000. The green spots represent FAM-labeled miRNAs and the nucleus is stained with DAPI (blue). AE2G3 in complex with miR155 **(A)** and BDEF33 in complex with miR155 **(B)** represent cellular uptake of labeled dendrimeres complexes; while non-labeled dendrimeres AE2G3 **(C)** and BDEF33 **(D)** as well as miR155 alone **(E)** didn't differ from the negative control **(F)**. Lower cases **(a-f)** represent the correspondent phase-contrast images on the magnitude X400 where the cytoplasm is indicated by arrows.

Figure S2. Evaluation of proper toxic effects of cationic carbosilane (BDEF33) and phosphorus (AE2G3) dendrimers of the third generation on PBMCs viability in comparison with non-treated control cells (NTC). Mann-Whitney test was used. Letters mark significant differences ($p<0.05$): “a” – in comparison with NTC, “b” – in comparison with treatment in concentration 0.3 µM, “c” – in comparison with treatment in concentration 1 µM, “d” – in comparison with treatment in concentration 3 µM, “e” – in comparison with treatment in concentration 10 µM, “f” – in comparison with treatment in concentration 30 µM. Bar show the significant difference between two variants of complexes.

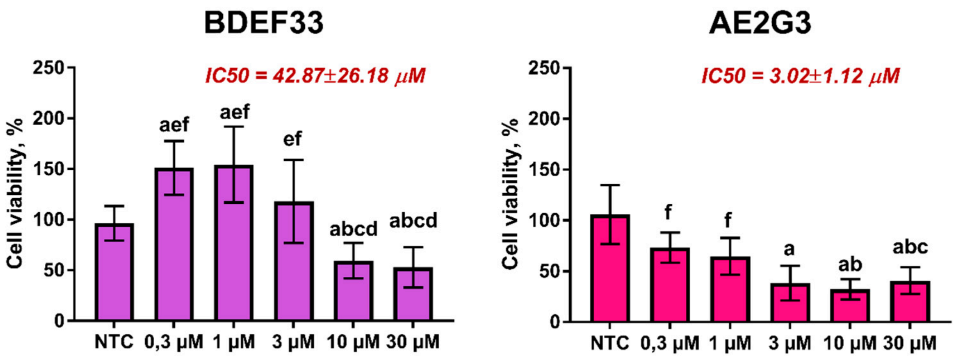

For both dendrimers dose-dependent toxic effect was observed for medium and high concentrations. Interestingly, cell viability was increased in comparison with NTC after treatment by BDEF33 in smaller concentrations – 0.3 µM ($p=0.009$) and 1 µM ($p=0.021$). For AE2G3 cell viability curve looks more plane.

AE2G3 was found to be more toxic for PBMCs as BDEF33 being taken in low and medium concentrations 0.3 µM ($p=0.005$), 1 µM ($p=0.0076$), 3 µM ($p=0.007$). These data also correlate with the values of IC50 for the dendrimers: IC50 of AE2G3 is much lower (3,02±1,12 µM) than IC50 of BDEF33 (42,87±26,8 µM).

Figure S3. Evaluation of dendriplexes toxicity in comparison with non-treated control (NTC) and mock control (free dendrimer in the highest concentration being used – 1,31 μM for BDEF33 and 0.66 μM for AE2G3). Mann-Whitney test was used.

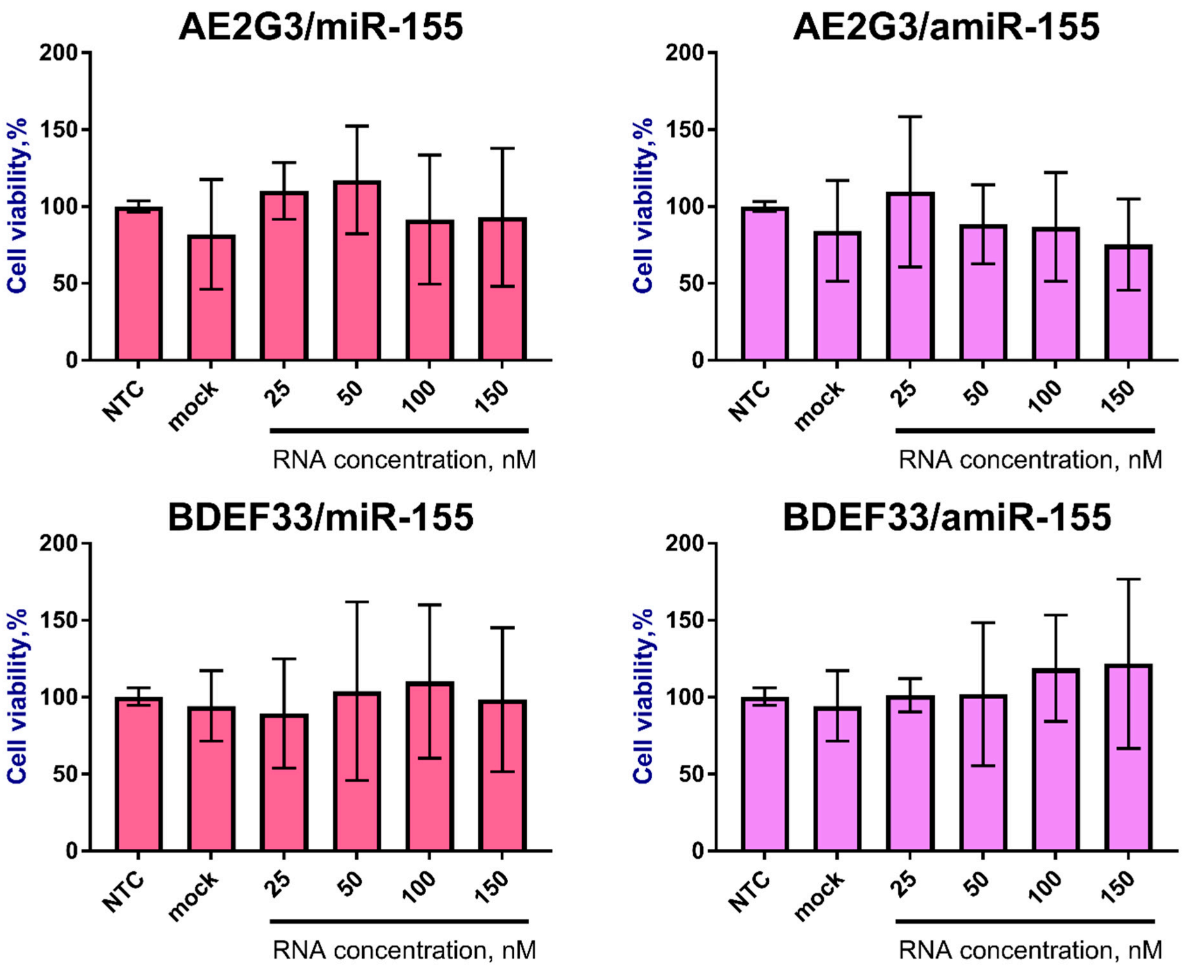

Figure S4. Evaluation of LDH activity after dendriplexes treatment (10 min, 20 min) in comparison with non-treated control (NTC), free miRs and free dendrimers. Mann-Whitney test was used. Letters mark significant differences ($p<0.05$) between groups: “a” – in comparison with NTC; “b” – in comparison with a free dendrimer; “c” – in comparison with a free miR; “d” – in comparison with similar BDEF33-based complex; “e” – in comparison with similar AE2G3-based complex. Bars show the significant differences between columns in the subgroups.

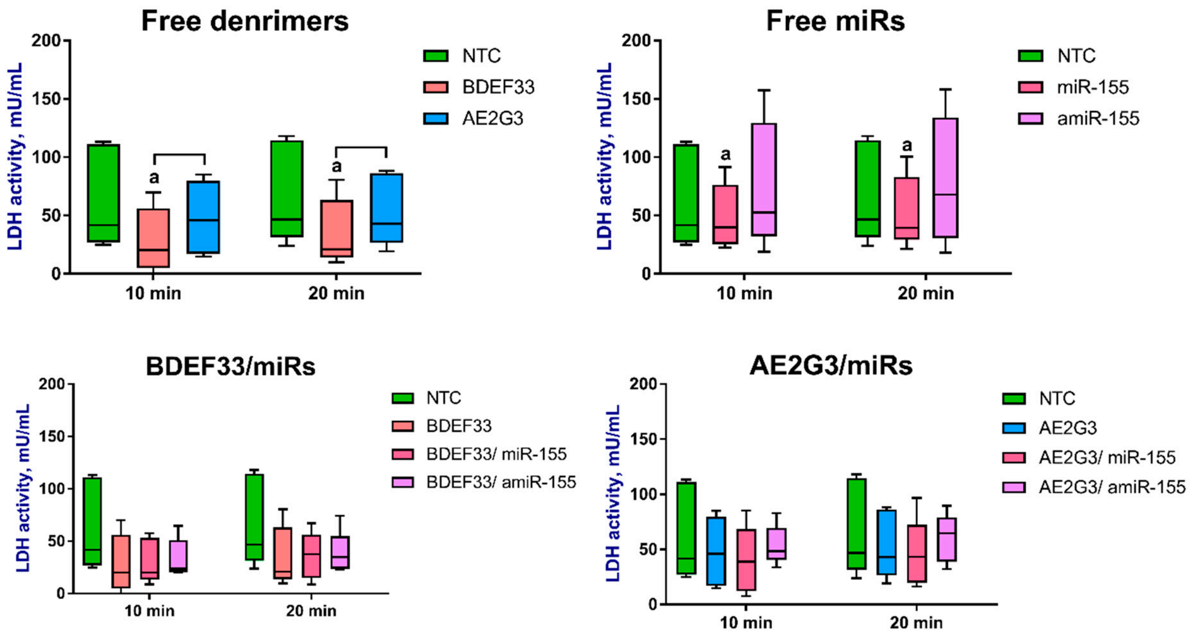

Figure S5. Evaluation of apoptosis parameters in CD4+ and CD8+ subsets after treatment by free dendrimers, free miRs or dendriplexes in comparison with non-treated control (NTC). Mann-Whitney test was used.

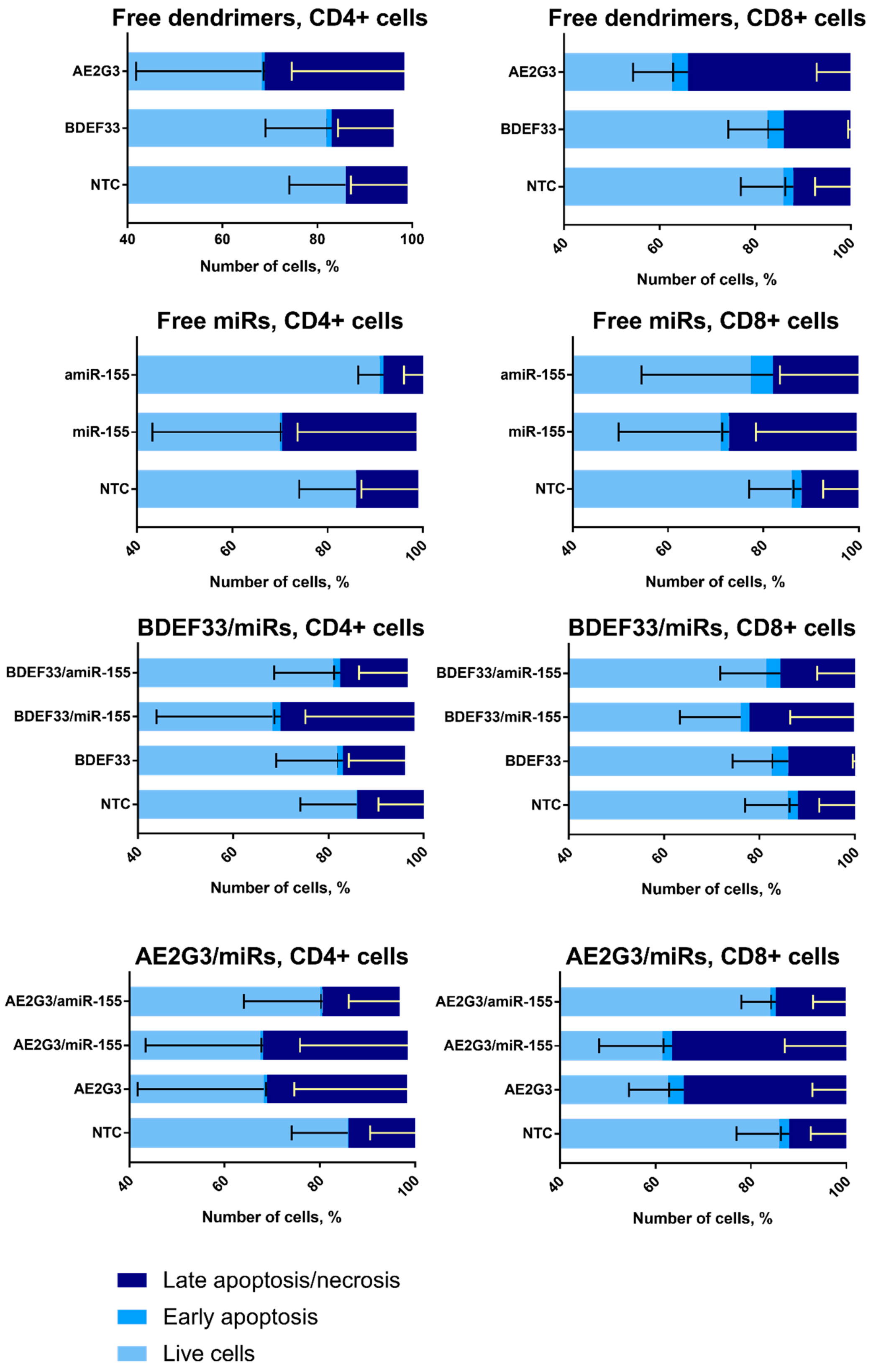

Figure S6. Evaluation of subsets percentage after the treatment by dendriplexes or their components (72 h): $CD3^+$, $CD4^+$, $CD8^+$. Mann-Whitney test was used.

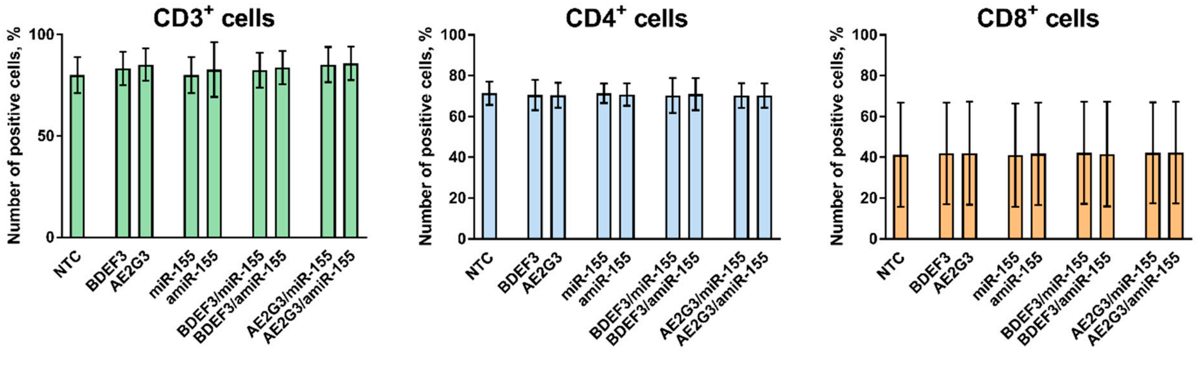

Figure S7. Evaluation of T-cells subsets proliferation after the treatment by dendriplexes or their components (72 h). Mann-Whitney test was used.

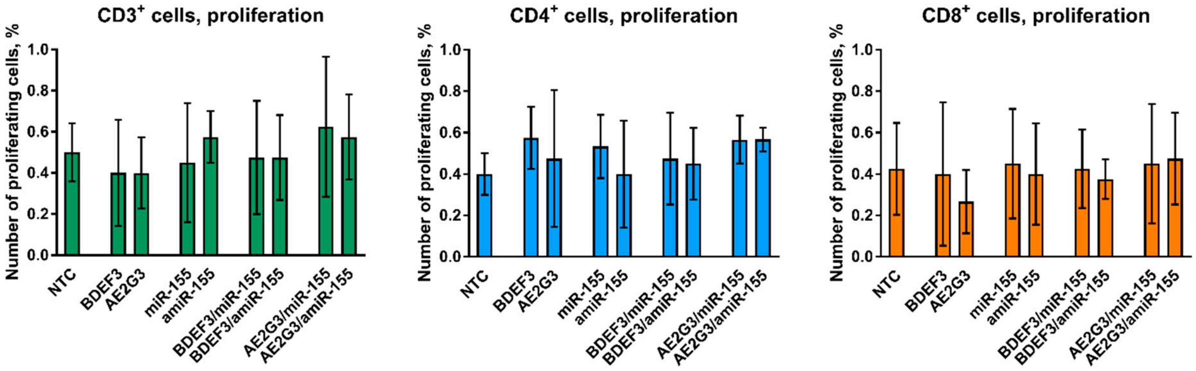

Figure S8. Evaluation of expression of CD25 activation marker on CD4+ and $CD8^+$ T-lymphocytes after 72 h of treatment by dendriplexes or their complexes. Mann-Whitney test was used. Letters mark significant differences ($p<0.05$): “a” – in comparison with NTC, “b” – in comparison with treatment by free dendrimer, “c” – in comparison with treatment by free miR, “d” – in comparison with treatment by BDEF33-based complex, “e” – in comparison with by AE2G3-based complex.

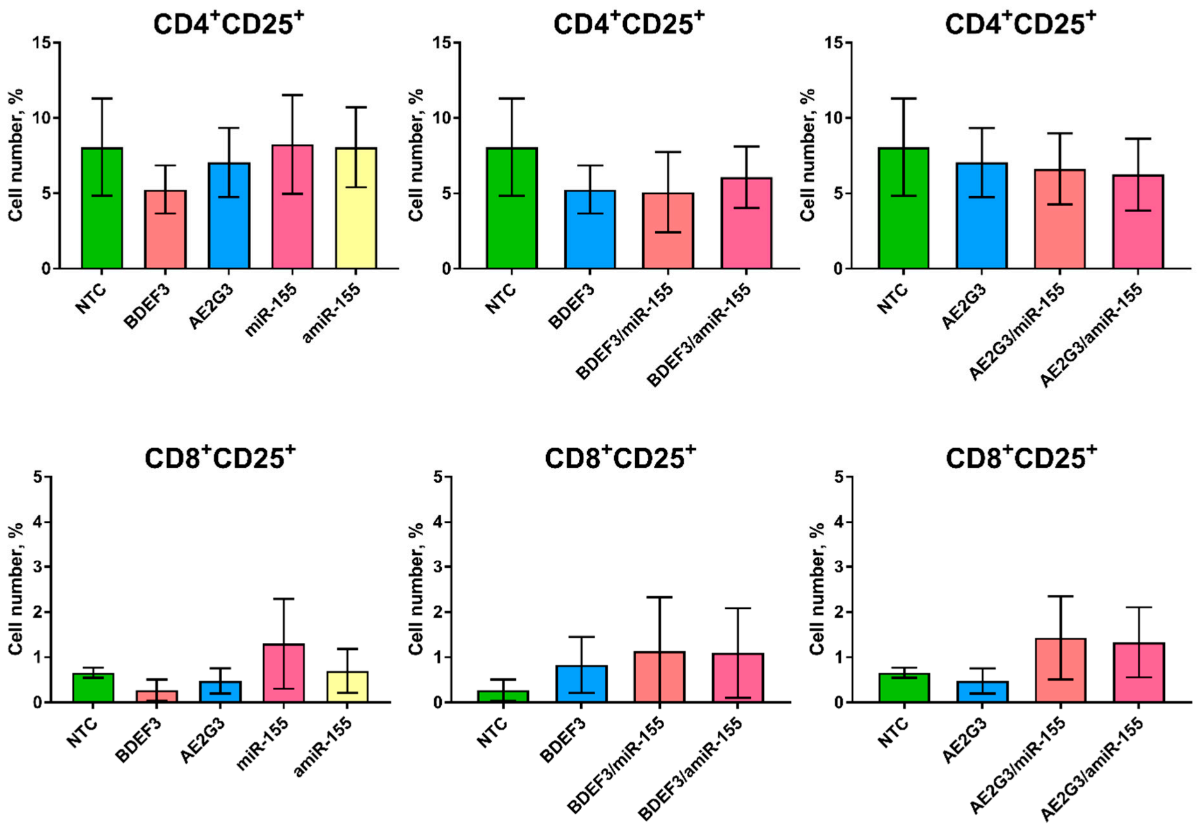

Figure S9. Evaluation of expression of granzyme B (GrzB) and perforin (Perf) by cytotoxic CD8⁺ T-lymphocytes and NK-cells (CD16⁺CD56⁺) after treatment by dendriplexes or their components. Mann-Whitney test was used.

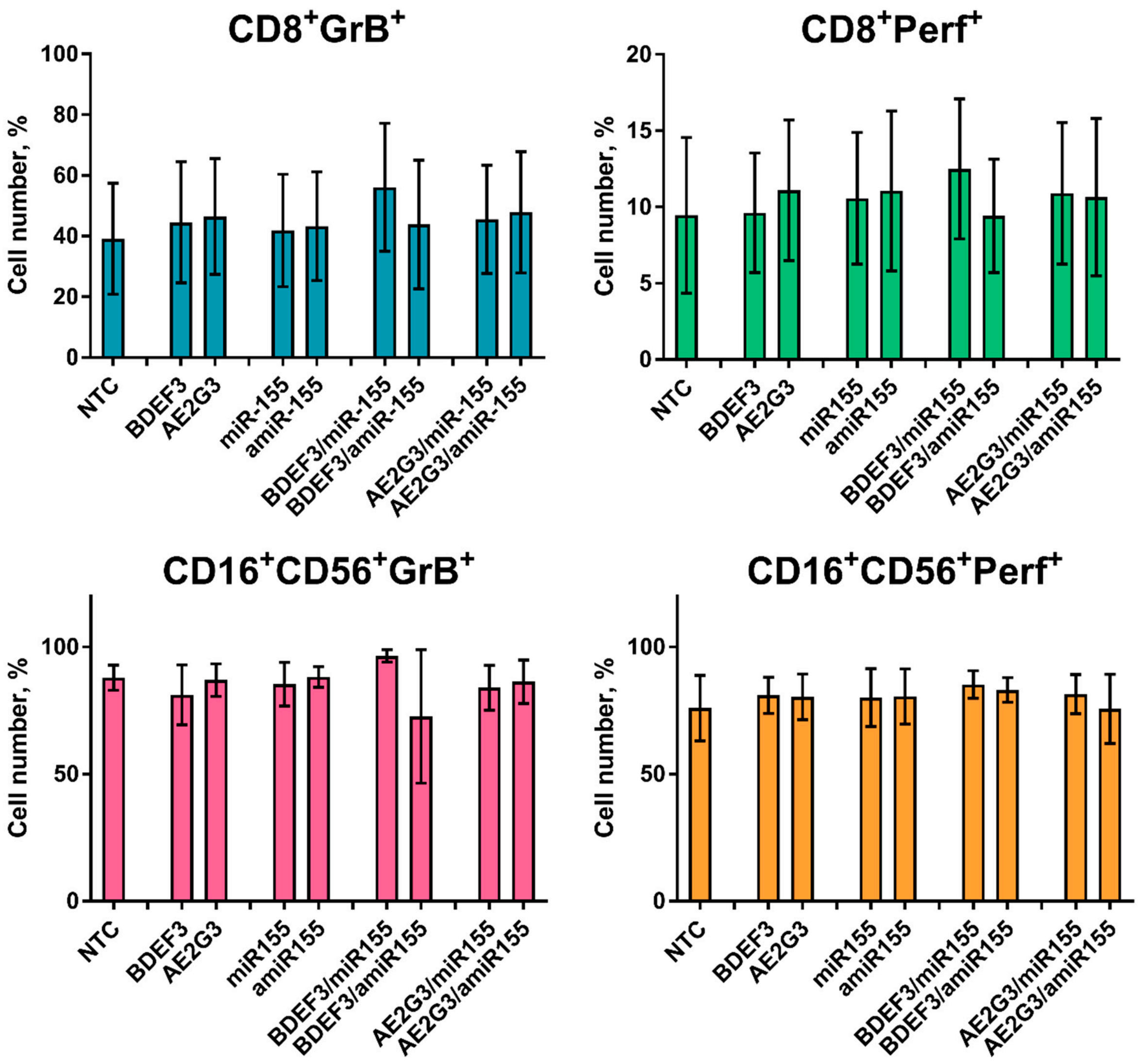

Supplement: Supplementary file 1 [file pharmaceutics-15-00148-s001.zip › pharmaceutics-2073117-supplementary.pdf]
